# Multifocal Aeromonas Osteomyelitis in a Child with Leukemia

**DOI:** 10.1155/2016/8159048

**Published:** 2016-04-21

**Authors:** Dimitrios Doganis, Margarita Baka, Maria Tsolia, Apostolos Pourtsidis, Evangelia Lebessi, Maria Varvoutsi, Despina Bouhoutsou, Helen Kosmidis

**Affiliations:** ^1^Oncology Department, “P. & A. Kyriakou” Children's Hospital, Thivon & Levadias Street, 11527 Athens, Greece; ^2^Second Department of Pediatrics, National and Kapodistrian University of Athens School of Medicine, “P. & A. Kyriakou” Children's Hospital, Thivon & Levadias Street, 11527 Athens, Greece; ^3^Department of Microbiology, “P. & A. Kyriakou” Children's Hospital, Thivon & Levadias Street, 11527 Athens, Greece

## Abstract

*Aeromonas hydrophila* is a Gram negative organism causing both intestinal and extraintestinal disease. The case of a 14-year-old girl with underlying immunodeficiency and leukemia who developed systemic* A. hydrophila* infection is described in this report. While in deep bone marrow aplasia she developed fever, severe pain in the lower extremities, and swelling of the left femur. Blood culture showed* Escherichia coli* and* A. hydrophila* whereas pus culture from the soft tissue swelling showed the presence of* A. hydrophila*. Imaging studies showed diffuse osteolytic lesions. Patient received 5 months of intravenous and oral antibiotics and she improved clinically whereas the radiology findings persisted.

## 1. Introduction

*Aeromonas* species are Gram negative bacteria commonly found in many food samples or a variety of aquatic environments [1–3]. These organisms may be isolated from healthy carriers and from patients suffering from diarrhoea as well. In a study from Greece,* Aeromonas* spp. were identified in 7% of children with acute diarrhoea [4] whereas authors from Spain have demonstrated that about 1% of healthy adults are carriers [5].

Human diseases caused by* Aeromonas* spp. can be classified into two major groups: septicemia and gastroenteritis [1].* Aeromonas* spp. infection may also present with osteomyelitis, ocular infections, myositis, meningitis, endocarditis, hemolytic uremic syndrome, and peritonitis. Although* Aeromonas* spp. infections have been reported in healthy children, the most clinically significant of them should be considered in immunocompromised patients such as the febrile neutropenic cancer patients [2, 3, 6, 7]. The case of multifocal* A. hydrophila* osteomyelitis with extensive involvement of both lower limbs in a 14-year-old girl with immunodeficiency and leukemia is presented in this report.

## 2. Case Presentation

**Figure 1:**
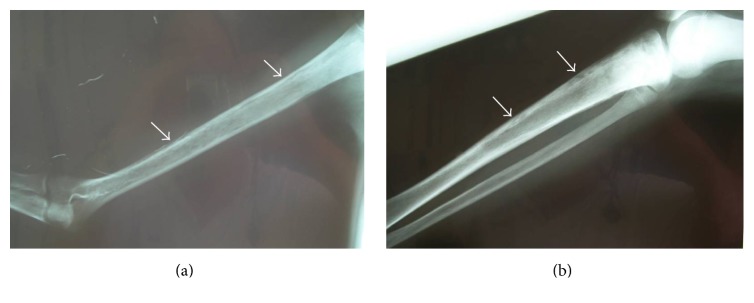
Diffuse osteolytic lesions in the right humerus ((a), arrows) and right tibia ((b), arrows).

A 14-year-old Roma girl with a known history of immunodeficiency (hypogammaglobulinemia) and family history of cancer and immunodeficiency was admitted to our department. No severe infections were reported in the past. Our patient presented with history of bone pain lymphadenopathy, dental carries, and fever and the diagnosis of acute nonlymphocytic leukemia M0 as per FAB classification was made. She was started on chemotherapy according to BFM 98 protocol and achieved remission after the 1st induction course. Three months since the onset of treatment, during consolidation therapy and while deeply aplastic (WBC: 400/mm^3^, Hb: 7,9 gr/dL, and PLT: 20000/mm^3^), she developed severe pain in the lower extremities, fever with chills (39°C), and swelling of the lower third of the left femur whereas on examination she had pain on passive movement. The CRP value was 454 mg/L. A bone scan was performed and revealed irregular uptake in the acetabulum and in the lower extremities and the right humerus, indicative of bone infiltration. Radiographs were initially normal but when repeated later revealed diffuse and extensive osteolytic lesions in the acetabulum, the long bones of both lower extremities, and the right humerus (Figure 1).

Blood cultures, performed by Bactec 9240 system (Becton Dickinson, USA), showed two microorganisms,* Escherichia coli* and* A. hydrophila* (lactose, nonfermenting), as identified by Vitek 2 (BioMerieux SA, France). Gram stain of the pus from the soft tissue swelling demonstrated Gram negative bacilli. Culture of pus on blood agar plate demonstrated large, opaque, beta-hemolytic colonies resembling* Aeromonas* spp., whereas on MacConkey's agar plate two types of colonies grew (lactose, fermenting and nonfermenting, both oxidase positive). Vitek 2 was used and a battery of biochemical tests were performed in order to identify the two different types of organisms to the species level as* A. hydrophila* [8]. All* A. hydrophila* isolates from blood and pus were tested to antibiotics by Vitek 2 System and by disc diffusion method according to CLSI guidelines [9]. Although the two strains from pus were phenotypically different, they showed the same susceptibility pattern: susceptible to cefotaxime, ceftriaxone and ceftazidime (mic ≤ 1 mg/L), piperacillin/tazobactam (mic = 16/4 mg/L), gentamicin and tobramycin (mic ≤ 1 mg/L), amikacin (mic ≤ 2 mg/L), and ciprofloxacin (mic ≤ 1 mg/L), intermediately susceptible to amoxicillin/clavulanate (mic = 16/8 mg/L), and resistant to ampicillin (mic ≥ 32 mg/L) and sulfamethoxazole-trimethoprim (mic ≥ 320 mg/L). Exactly the same susceptibility pattern had been found concerning the blood* A. hydrophila* isolate.

Our patient was initially treated with ceftazidime (IV, 50 mgr/Kgr/8 h for 8 weeks) and gentamicin (IV, 2 mgr/Kgr/8 h for 5 weeks). Later in the course, within the same period since the beginning of antibiotic treatment, teicoplanin (IV, 10 mgr/kgr/day for 2 weeks) and liposomal amphotericin (IV, 3 mgr/Kgr/day for 2 weeks) were added to the regimen because of persistent fever. Overall, our patient received 3 months of IV antibiotics (2 months initially, plus 1 month of treatment with ceftriaxone IV, 100 mgr/Kgr/day for 1 month) followed by 2 months of oral ciprofloxacin (15 mgr/Kgr/12 h) as well as IV *γ*-globulin monthly.

During her hospitalization she refused to walk, initially due to severe pain and later due to muscular weakness from prolonged immobilization. Fever persisted for 3 months; her active movements were improved whereas the radiology findings persisted. She also developed severe osteoporotic changes and was treated with bisphosphonates, calcium, and vitamin D. Therapy for her underlying leukemia had to be modified. Two months later, her leukemia relapsed and she succumbed to her disease progress.

## 3. Discussion

We described a girl with underlying immunodeficiency and leukemia who developed* A. hydrophila* infection presenting with septicemia and multifocal, disseminated osteomyelitis and, to the best of our knowledge, this is the first case of multifocal* Aeromonas* spp. infection in a leukemic child.

*A. hydrophila* is considered as an opportunistic pathogen, but it has been increasingly isolated from both healthy and immunocompromised subjects [2]. Infections caused by this organism in children are far less common compared to adults [3].

*Aeromonas* species cause both intestinal and extraintestinal disease.* A. hydrophila* is the most common species isolated in patients with extraintestinal disease, followed by* Aeromonas sobria*. Bacteremia, skin, and soft tissue infections are the commonest manifestations of extraintestinal disease and are mainly attributed to* A. hydrophila* [10, 11]. The most common clinical presentation is cellulitis with a good prognosis if bacteremia is not present. Septic arthritis due to* A. hydrophila* may also occur but it is less common [12, 13]. Necrotizing fasciitis from* Aeromonas* with extensive subcutaneous and muscle necrosis is a rapidly progressive and potentially fatal infection in children who have underlying systemic diseases or immune dysfunction [14].

Infection by this organism has been associated with water-related injuries or penetrating wound trauma [15]. Unlike reports that have described* A. hydrophila* infection after exposure to aquatic environment, there was no such history in our patient. In a previously published report, an 11-year-old immunocompromised child developed cellulitis and abscess due to* A. hydrophila* at the site of bone marrow aspiration after swimming in a freshwater lake [16] whereas other authors reported cases of bone infection due to* A. hydrophila* in a complex fracture or in an implant [17].* Aeromonas* spp. infections of the hepatobiliary or pancreatic system have also been reported and cholangitis is the most common manifestation among these cases [18].

Our patient presented with bacteremia due to* A. hydrophila* and simultaneously with osteomyelitis due to two phenotypically different strains of* A. hydrophila*, one of them phenotypically identical to the blood* A. hydrophila* isolate.* Aeromonas* bacteremia is most likely to occur in patients with acute leukemia and there is a predominance of male patients [19]. The case fatality rate for infants and children with* A. hydrophila* septicemia is high and reaches approximately 50% and is probably associated with severe immunosuppression of these patients [20].

When osteomyelitis from* A. hydrophila* is suspected, plain films are mandatory and when these films are negative, skeletal scintigraphy is indicated. MRI should be performed when symptoms are localized to the pelvis or pelvic osteomyelitis is indicated by scintigraphy in order to look for abscesses [21]. Not unexpectedly, when our patient first presented with pain, fever, and swelling in the extremities bone scintigraphy revealed diffuse lesions while skeletal X-rays were normal.

Concerning the treatment of extraintestinal infection due to* Aeromonas*, this is similar to Gram negative infections. Especially for bacteremia due to* Aeromonas* species, the combination of an aminoglycoside and a cephalosporin is the appropriate therapy [19] whereas the duration of treatment depends on the site of infection and the response to antibiotics [3].

Our patient with underlying immunodeficiency and leukemia developed* Aeromonas* infection and presented with septicemia and severe, diffuse, and multifocal osteomyelitis leading to severe and irreversible handicap and was successfully treated with combination of antibiotics for a prolonged period of time. Conclusively, the diagnosis of osteomyelitis should be highly suspected when fever, neutropenia, and bacteremia are accompanied by unexplained pain in the extremities and, therefore, extensive imaging evaluation, biopsy, and cultures from the lesions are needed. Prompt diagnosis and early antibiotic treatment can improve the outcome of this serious infection especially in immunocompromised population.
